# Presence of Methicillin-Resistant Staphylococci and Carbapenemase-Positive *Acinetobacter* Isolates on Surfaces in German Dog Daycare Facilities and Correlation with Cleaning Practices

**DOI:** 10.3390/vetsci11110568

**Published:** 2024-11-15

**Authors:** Stephanie Forbes, Ellen Prenger-Berninghoff, Christa Ewers, Maren Doelle, Anja Roethig

**Affiliations:** 1 AniCura Tierärztliche Spezialisten Hamburg, Rodigallee 85, 22043 Hamburg, Germany; maren.doelle@anicura.de; 2 Institute of Hygiene and Infectious Diseases of Animals, Faculty of Veterinary Medicine, Justus Liebig University Giessen, Frankfurter Straße 87-89, 35392 Giessen, Germany; ellen.prenger-berninghoff@vetmed.uni-giessen.de (E.P.-B.); christa.ewers@vetmed.uni-giessen.de (C.E.); 3 Tierklinik Neu-Isenburg, Carl-Friedrich-Gauß-Straße 5, 63263 Neu-Isenburg, Germany; a.roethig@tierklinik-neu-isenburg.de

**Keywords:** antimicrobial resistance, dog daycare facilities, methicillin-resistant staphylococci, *Acinetobacter* species, transmission, risk factors, one health

## Abstract

Many bacteria, including methicillin-resistant staphylococci and opportunistic pathogens such as *Acinetobacter* (*A*.) species, in particular members of the *A. calcoaceticus*—*A. baumannii* (*Acb*) complex, are known to survive in environmental settings. This increases the risk of bacterial spreading and transmission to animals and humans, especially in institutions with a high animal population density. This study aimed to identify the presence of multidrug-resistant (MDR) staphylococci and *Acinetobacter* species in dog daycare facilities (DDFs). The surfaces of 16 DDFs were sampled and information about cleaning practices was obtained. After microbial identification, the detected isolates were investigated by multiplex PCR for antimicrobial resistance genes. In 200 location samples, 38 staphylococci and 109 *Acinetobacter* spp. isolates were identified. Methicillin-resistant genes were confirmed in 18 staphylococci isolates from four DDFs, including *Staphylococcus* (*S.*) *equorum*, *S. saprophyticus*, *S. cohnii*, *S. lentus*, and *S. haemolyticus*. Eight *A. radioresistens* isolates comprising the *bla*_OXA-23_ carbapenemase gene and seventeen isolates belonging to the *Acb* complex were also isolated. This is the first investigation for MDR pathogens in DDFs, a close human–animal interaction environment. Though the role of DDFs in the transmission and spreading of MDR bacteria is not known, the findings should contribute to the public awareness and underscore the necessity of adequate cleaning protocols.

## Simple Summary

This study focused on understanding whether putative pathogenic bacteria with resistance to common antibiotics are present in dog daycare facilities and how these bacteria might spread. Such pathogens can be difficult to treat and may pose a risk to both animals and humans. Sixteen different dog daycare facilities were included where bacterial screening was performed on various surfaces, such as dog beds, floors, and bowls. The bacteria found included 18 methicillin-resistant staphylococci, 8 carbapenemase-positive, and 17 *Acinetobacter* isolates with a high risk of developing resistance. Although most of those bacteria were of an opportunistic nature, thus revealing a low pathogenic potential for human and animal health, they could pose a risk by passing their antimicrobial resistance determinants to other, more harmful bacteria. The study also looked at how these facilities clean their accommodations and found that the current cleaning practices are not always effective in removing these bacteria. This leads to the conclusion that better cleaning guidelines are needed in dog daycare facilities to prevent the spread of antibiotic-resistant bacteria. This research is valuable to society because it highlights a potential health risk in places where many animals are kept together and emphasizes the importance of good hygiene to protect public health.

## 1. Introduction

Under the pressure of antimicrobial selection caused by the usage of multiple antimicrobial agents against pathogenic bacteria, the presence of antimicrobial resistance (AMR) in human and veterinary medicine has been a rising concern for decades. Bacteria are able to acquire resistance genes and mobile genetic elements which can be transferred to other bacteria of the same or different genus. This results in certain classes of antibiotics being less or no longer effective, leading to treatment failures in patients. The infections caused by these bacteria are challenging and have become a significant public health threat. Additionally, these pathogens show a greater ability to proliferate in animals, humans, and the environment [1,2]. In 2012, a grading system was defined by Magiorakos et al. (2012) to better describe the severity of AMR. One of the most common antimicrobial resistance profiles includes the term “multidrug-resistant” (MDR). Bacteria classified as MDR are resistant to at least one agent of three or more antimicrobial categories, which often includes a key antimicrobial agent demonstrating cross- or co-resistance to multiple classes of antimicrobials. This can be, for example, methicillin resistance in staphylococci [2].

In veterinary medicine, methicillin-resistant *Staphylococcus* (*S.*) *pseudintermedius* (MRSP) represents one of the most dominant MDR pathogens in canine skin and soft-tissue infections [3]. These bacteria carry the *mecA* gene, which encodes the penicillin-binding protein PBP2a. High-level methicillin resistance is caused subsequently by the crosslinking of the bacterial cell walls, leading to unresponsiveness to all β-lactam antibiotics. [4]. Methicillin resistance can also affect many other staphylococci species like *S. aureus*, *S. schleiferi*, *S. epidermidis*, and *S. haemolyticus.* Staphylococci are subdivided according to the presence of the virulence factor coagulase into coagulase-positive staphylococci (CoPS) and coagulase-negative staphylococci (CoNS). The more clinically relevant CoPS like *S. pseudintermedius* colonize canine skin frequently, whereas CoNS are considered as transient commensals as well as opportunistic pathogens. In the case of a cutaneous or systemic disease, skin surface defense mechanisms are disrupted and this leads to skin infection (bacterial pyoderma) or otitis externa. Treatment often includes the local or systemic administration of antimicrobial agents, which may be less or not effective if the infection is driven by a methicillin-resistant staphylococcus (MRS) [3]. Recent studies have shown that even after the healing of the disease, many dogs still reveal MRSP carriage, which can last for more than one year [5,6]. These isolates can then be transmitted to contact dogs and the environment via dog beds, bowls, and floors in households [5,7]. Spreading is also possible in pet grooming salons, where MRS species have been detected on clipper blades, clipper handles, and leashes [8].

In 2017, the World Health Organization (WHO) published a list of global priority pathogens, including 12 bacterial species classified as having the risk of critical, high, or medium antibiotic resistance. *Acinetobacter* (*A.*) *baumannii* was the first to be mentioned [9]. *Acinetobacter* spp., especially members of the *A. calcoaceticus*—*A. baumannii* (*Acb*) complex, namely *A. calcoaceticus*, *A. baumannii*, *A. pittii*, and *A. nosocomialis* are opportunistic pathogens associated with a wide range of infections in humans and animals. Several *Acinetobacter* spp. are intrinsically resistant to different antibiotic classes and additionally acquire AMR determinants, including those conferring resistance to “last resort” antibiotics in human medicine, such as carbapenems. Antimicrobial resistance is induced by acquired carbapenemases such as OXA-23. *Acinetobacter* species harboring a carbapenemase gene show an increased expression of efflux pumps and oxacillinases, capable of removing antimicrobial agents [10]. Clinically, they are involved in respiratory and urinary tract infections, as well as wounds and abscesses [11]. However, during clinical outbreaks, patients are presented with an underlying disease [12]. Limited therapeutic options often lead to life-threatening outcomes with an increasing mortality rate in patients [9,11]. Also in companion animals, multiple outbreaks, mainly due to *A. baumannii*, have been reported, including nosocomial spread within clinics and patient-to-patient transmission [12,13,14,15,16]. *Acinetobacter* species are highly environmentally persistent [12]. Especially, hospital settings seem to be a potential reservoir for MDR *A. baumannii* as apparent from a prolonged survival, and these facilities show an increased percentage of isolates. Also, clinical devices and medical staff play a significant role in pathogen transmission, and studies show that *Acinetobacter* isolates can be found on curtains, mattresses, pillows, urinals, sinks, and door handles [13,17].

Antimicrobial resistance is one of today’s greatest public health threats, and the “One Health” concept emphasizes that not only humans are affected, but also animals and the environment. As companion animals and their owners often share the same habitat, the interaction of pathogens reflects this close relationship between humans and animals [1,18]. Studies have confirmed that dogs and their owners can carry the same staphylococci strains, suggesting a risk of the transmission of staphylococci from dogs to humans or vice versa [19,20]. Furthermore, it is known that multiple *A. baumannii* isolates from companion animals (dogs, cats, and horses) and the surfaces of veterinary hospitals actually belong to human clonal lineages, which caused outbreaks in human medicine [13,21]. Consequently, the transmission of pathogens between dogs and humans should not be underestimated.

Due to their clinical relevance and their spreading potential, various risk factors predisposing animals to an infection with MRSP, or carbapenemase-resistant *Acinetobacter* spp., have been identified. Those included veterinary visits, hospitalization, and clinical interventions, highlighting the importance of a contaminated clinical environment as a key factor for transmission [12,22].

Locations with a high animal population density such as veterinary institutions, animal shelters, grooming salons, and dog daycare facilities (DDFs) may potentially be sources of MDR pathogens. However, data concerning the presence of pathogenic and antimicrobial-resistant bacteria in DDFs are lacking.

The objective of this study was to evaluate the presence of MDR bacteria in DDFs and their potential role in spreading these pathogens amongst the dog population. Therefore, the incidence of MRS on different surface areas in DDFs was investigated. As in the early part of the study, *Acinetobacter* species were identified in more than one DDF, and due to their relevance in the “One Health” agenda, we decided to include these global priority pathogens in our study and in the statistical analysis as well. Furthermore, information regarding cleaning procedures in DDFs was obtained, as we hypothesized that inadequate cleaning protocols could be associated with the spread of MDR bacteria.

## 2. Materials and Methods

### 2.1. Participating Dog Daycare Facilities

In this prospective, cross-sectional study, DDFs, located in northern and central Germany, were recruited by an informative email flyer and telephone inquiries. Included were such daycares where dogs were picked up daily as well as boarding kennels that allow overnight stays of several days. All the facilities used kennels, housing either one or multiple dogs; open areas (indoor or outdoor or a combination); and a drop-off/pick-up area. For sampling, DDFs were visited once at an unscheduled time. During the visit, the DDF’s manager was asked to fill in a questionnaire about client capacity, daily occupancy, and cleaning protocols. The detailed questionnaire is presented in Supplementary Materials.

### 2.2. Sample Collection

All the DDFs were sampled once between July 2022 and July 2023, with 6–8 samples being taken depending on the availability of the sample locations. Those were grouped into: dog beds (including the surfaces of beds, blankets, pillows, and couches), floors (including floors, stairs, and carpets that were walked on regularly), doors (including door surfaces, door handles, and dog safety gates), water taps (including the outlet, the washbasin, its drainage, and laundry tables), leashes (leashes and muzzles, focusing on areas that frequently come into contact with the skin of dogs and handlers), dog transport boxes (inner surfaces), and bowls (including margin and bottom). All the samples were taken wearing nonsterile nitrile gloves (multi-com GmbH & Co., KG, Ahrensburg, Germany) which were changed between each sample site. Each location was sampled twice by placing a square template of 10 × 10 cm on the location and swabbing it once with a dry and once with an isotonic sodium chloride solution (B. Braun Melsungen AG, Melsungen, Germany)-moistened sterile swab (Transystem, Copan, Brescia, Italy). Afterwards, the samples were submitted to the Institute of Hygiene and Infectious Diseases of Animals, Justus Liebig University Giessen, for bacterial investigation and further analysis.

### 2.3. Isolation and Identification of Bacterial Isolates

The sampled swabs from the first three DDFs were streaked on standard nutrient agar (Oxoid, Wesel, Germany) containing 5% defibrinated sheep blood, on water-blue metachrome-yellow lactose agar according to Gassner (Sifin Diagnostics GmbH, Berlin, Germany), and on oxacillin-resistant screening agar (Oxoid, Wesel, Germany). The plates were incubated at 37 °C in ambient air and analyzed after 24 and 48 h. Due to low recovery rates for staphylococci and the fact that broad culturing identified a high prevalence of *Acinetobacter* species, the study was expanded. The sampled swabs from the following DDFs were additionally placed in standard I nutrient broth (E. Merck KG, Darmstadt, Germany) for bacterial enrichment and were incubated overnight at 37 °C. The overnight cultures were then streaked on the same media as stated above and incubated at 37 °C for 24 h. Morphologically different colonies were subcultured on standard nutrient agar containing 5% defibrinated sheep blood and bacterial identification was carried out by matrix-assisted laser desorption time-of-flight mass spectrometry (MALDI TOF MS; Bruker Daltonics, Bremen, Germany) applying the standard MBT Compass reference library (version 10.0.0.0). The isolates were stored in brain heart infusion broth with 30% glycerol (Oxoid, Wesel, Germany) at −70 °C.

### 2.4. Genotypic Investigations

All the staphylococci isolates were investigated for the presence of AMR genes *mecA* and *mecC* by using a multiplex PCR that additionally amplifies a gene specific for the species *S. pseudintermedius*. [23,24] DNA was prepared utilizing the lysis by the boiling method. One reaction mixture for the PCR consisted of 3 µL DNA in a total volume of 30 µL composed of 0,03 U DreamTaq DNS Polymerase (Thermo Fisher Scientific, Dreieich, Germany), 1 µM of each primer, 133 µM deoxynucleoside triphosphate mixture (Rapidozym, Berlin, Germany), and one reaction buffer (Thermo Fisher Scientific, Dreieich, Germany). The reaction mixture was thermally cycled once at 94 °C for 5 min, 30 times at 94 °C 30 s, 56 °C 30 s, and 72 °C 1 min, and final elongation at 72 °C for 5 min. All the *Acinetobacter* spp. isolates were screened for β-lactamase genes coding for carbapenemases of the families OXA-23/-40/-58/-143/-153, VIM, NDM, KPC, and OXA-48 by PCRs utilizing previously published primers and protocols [15]. The PCR methods listed are well established with a high specificity and sensitivity and have been published several times [15,23,25].

### 2.5. Statistical Methods

Binomial tests were used to evaluate the frequency of the isolates. A chi-squared proportion test analyzed the differences in the bacterial load between individual localizations, followed by multiple pairwise comparisons. These were adjusted with the Bonferroni correction to discriminate the localizations with significant differences. To investigate the correlation between the isolated species carrying AMR genes and the different sample locations, the isolates were identified, and a Pearson’s chi-squared test was performed. A contingency table examined the distribution of the sample results for each DDF.

## 3. Results

Ninety-one dog daycare facilities were approached for inclusion in the study. A total of 16 DDFs agreed to participate and were sampled, including 12 indoor DDFs, three boarding kennels, and one outdoor facility. A total of 200 samples were evaluated. Respectively, these included 59 swabs of dog beds, 54 swabs of floors, 15 swabs of doors, 16 swabs of water taps, 20 swabs of leashes, 12 swabs of dog transport boxes, and 24 swabs of bowls. There was a significant difference in the bacterial load (staphylococci and *Acinetobacter* isolates) between the individual localizations (*p* < 0.0001). Significantly more isolates (*p* < 0.05) were detected on the localization dog bed in comparison with transport boxes, leashes, water taps, and doors, as well as on the localization floor compared with transport boxes and leashes.

### 3.1. Detected Staphylococci and Acinetobacter spp. Isolates

**Figure 1:**
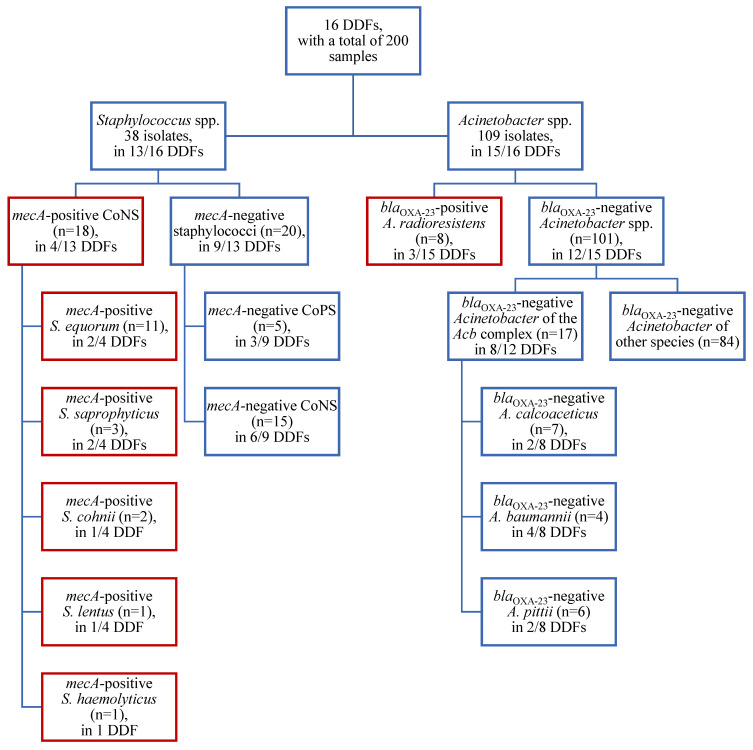
Isolation of *Staphylococcus* (*S*.) spp. and *Acinetobacter* (*A.*) spp. isolates, positive or negative, for the investigated antimicrobial resistance genes (*mecA*, *bla*_OXA-23_) from 16 dog daycare facilities (DDFs). Staphylococci species were divided into coagulase-positive and coagulase-negative staphylococci (CoPS and CoNS). For *Acinetobacter* spp., the *bla*_OXA-23_-negative *Acinetobacter* were summarized and the *A. calcoaceticus—A. baumannii* complex consisting of *A. calcoaceticus*, *A. baumannii*, and *A. pittii* was highlighted due to its clinical relevance.

**Figure 2:**
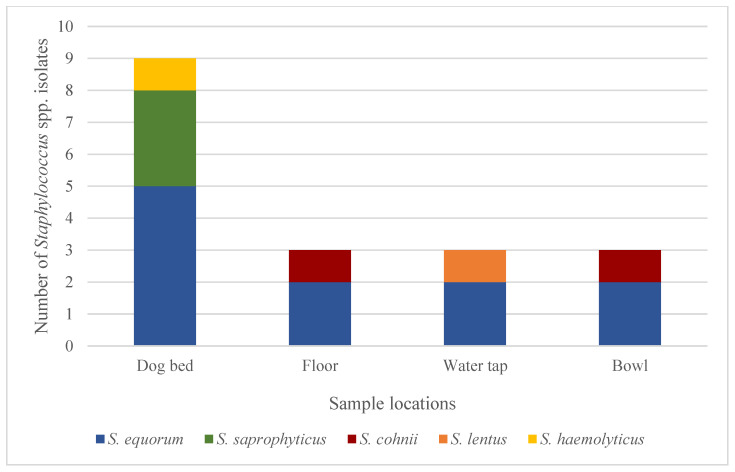
Identification of methicillin-resistant (*mecA*-positive) *Staphylococcus* spp. according to sample locations.

After evaluating all the samples, 38 isolates of staphylococci were identified in 13/16 DDFs (82%). Figure 1 provides an illustration of the number and detection of resistance genes of the staphylococci and *Acinetobacter* isolates, as well as the number of affected DDFs. Concerning the CoPS, *S. pseudintermedius* was identified at four sample sites in 2/16 different DDFs (13%). A single *S. aureus* isolate was detected in a bowl. The AMR genes *mecA* and *mecC*, conferring the MRS phenotype, could not be demonstrated in these five CoPS isolates. *Staphylococcus equorum*, as a member of the less virulent CoNS, was the most common isolated staphylococcus species (17/38 isolates, 45%). The *mecA* gene was identified in 18 CoNS isolates, which were obtained from four different DDFs. Those CoNS species comprised *S. equorum* (11/18 isolates, 61%), *S. saprophyticus* (3/18 isolates, 17%), *S. cohnii* (2/18 isolates, 11%), *S. lentus* (1/18 isolates, 6%), and *S. haemolyticus* (1/18 isolates, 6%). Methicillin-resistant isolates were most commonly detected in dog beds (9/18 isolates, 50%), followed by the floors (3/18 isolates, 17%), bowls (3/18 isolates, 17%), and water taps (3/18 isolates, 17%). The distribution pattern of the identified MRS is shown in Figure 2. All the other staphylococci isolates (*S. equorum* (6/38 isolates, 16%), *S. warneri* (3/38 isolates, 8%), *S. epidermidis* (1/38 isolates, 3%), *S. cohnii* (1/38 isolates, 3%), *S. xylosus* (1/38 isolates, 3%), *S. sciuri* (1/38 isolates, 3%), *S. fleurettii* (1/38 isolates, 3%), and one unidentified staphylococcus isolate (3%)) did not harbor the *mecA* or *mecC* gene.

**Figure 3:**
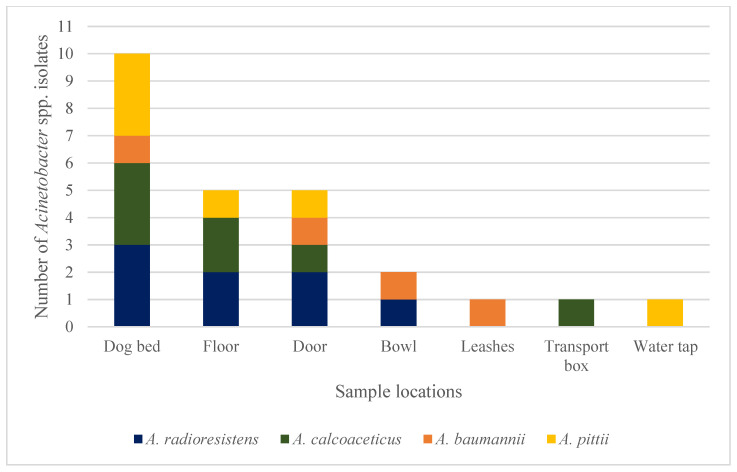
Identification of carbapenemase-positive *Acinetobacter* (*A*.) *radioresistens* and carbapenemase-negative *Acinetobacter* of the *A. calcoaceticus*—*A. baumannii* complex according to sample locations.

*Acinetobacter* spp. isolates were present in all but one DDF. A total of 109 isolates were obtained (Figure 1), including the species *A. johnsonii* (28/109 isolates, 26%), *A. lwoffii* (24/109 isolates, 22%), *A. radioresistens* (12/109 isolates, 11%), *A. calcoaceticus* (7/109 isolates, 6%), *A. pittii* (6/109 isolates, 6%), *A. albensis* (6/109 isolates, 6%), *A. baumannii* (4/109 isolates, 4%), *A. guillouiae* (4/109 isolates, 4%), *A. beijerinckii* (2/109 isolates, 2%), *A. parvus* (2/109 isolates, 2%), *A. ursingii* (2/109 isolates, 2%), *A. harbinensis* (1/109 isolates, 1%), *A. junii* (1/109 isolates, 1%), *A. towneri* (1/109 isolates, 1%), and nine unidentified *Acinetobacter* species (8%). All 17 isolates belonging to the *Acb* complex, namely *A. calcoaceticus*, *A. baumannii*, and *A. pittii* isolates, were negative for the investigated β-lactamase genes. They were identified in 7/16 DDFs (44%), most commonly isolated from dog beds (7/17 isolates, 41%), but also from the floor (3/17 isolates, 18%), door (3/17 isolates, 18%), and one isolate (6%) each from a bowl, leash, transport box, and water tap. Eight of the twelve *A. radioresistens* isolates revealed the presence of the carbapenemase gene *bla*_OXA-23_. These eight isolates were detected in three different DDFs at the following sample sites: dog beds (3/8 isolates, 38%), floor (2/8 isolates, 25%), doors (2/8 isolates, 25%), and bowl (1/8 isolates, 13%). Figure 3 shows the distribution pattern of the detected *Acinetobacter* isolates belonging to the *Acb* complex and the carbapenemase-positive *A. radioresistens* strains. None of the other β-lactamase genes that have been investigated could be identified in any of the isolates.

In one DDF, a *mecA*-positive *S. haemolyticus* was demonstrated simultaneously with an *A. calcoaceticus* isolate from a dog bed. In another DDF, not only MRS (*S. saprophyticus* and *S. cohnii*) but also one carbapenemase-positive *A. radioresistens* was detectable. Also, this isolate was obtained from a dog bed.

### 3.2. Cleaning Protocols of Dog Daycare Facilities

Information regarding cleaning practices and products applied in the DDFs was gathered through a questionnaire filled in by the DDF’s operators. Of the 16 participating DDFs, 15 institutions provided complete questionnaires. One DDF failed to complete the form and was excluded from this evaluation. The different cleaning practices and products are presented in Table 1. Cleaning frequency differed between once a day and once a month and was mainly performed by using commercial universal cleaning agents containing surfactants (10/15 DDFs). Four products also contained lactic acid. Other antimicrobial agents included alkyl-dimethyl-benzyl-ammonium-chloride (ADBAC), multiple alcohols, or acetic acid. Two DDFs used a product containing PermaZym^®^ technology (Biodor GmbH, Bocholt, Germany). There were no statistically significant correlations between the cleaning practices and the presence of MRS and *Acinetobacter* isolates tested positive for a carbapenemase gene.

## 4. Discussion

The first objective of this study was the detection of MRS on surfaces in DDFs because of their well-known potential as nosocomial pathogens in veterinary and human medicine. Thirty-eight staphylococci were identified in 81.3% (13/16) of the participating DDFs. Nearly half of the isolated staphylococci, distributed among four different DDFs (25% of the sampled DDFs), were *mecA*-positive (18/38). The MRS isolates could all be assigned to different CoNS species, with a predominance of *S. equorum* (n = 11), followed by *S. saprophyticus* (n = 3), *S. cohnii*, *S. haemolyticus*, and *S. lentus* (n = 1 each). The *mecA* gene is part of the *mec* gene complex, which is located on the staphylococcal cassette chromosome (*SCCmec*) [4]. Since the first identification of *S. aureus* in the early 1960s, resistance via the *mecA* gene has been confirmed in multiple other staphylococci species and has emerged as a worldwide challenge in human and veterinary medicine. *Staphylococcus pseudintermedius*, *S. schleiferi*, and *S. aureus* are the primary skin pathogens in small animals, with a prevalence of methicillin resistance between 0 and 45% for MRSP, 0 and 17% for MRSS, and 0 and 22% for MRSA, varying between studies [3]. In our study, none of these pathogens that frequently carry resistance genes were identified on surfaces in DDFs. However, the presence of *mecA* was detected in multiple isolates of species belonging to the group of CoNS.

*Staphylococcus epidermidis* and *S. haemolyticus* are the most common CoNS-causing nosocomial infections in humans and animals. They are opportunistic pathogens and are associated with skin, urinary tract, respiratory, and multiple other infections, mainly in immune-compromised patients [26,27]. In our study, one *mecA*-positive *S. haemolyticus* isolate was detected on a dog bed in one DDF and the presence of this strain could pose a risk to both immunosuppressed dogs and humans. *Staphylococcus lentus*, another CoNS, belongs to the *S. sciuri* group, which also includes *S. sciuri*, *S. vitulinus*, *S. fleurettii*, and *S. stepanovicii* [28]. Species from this group are commonly isolated from animals, humans, and the environment, but they are also associated with infections. *Staphylococcus lentus* isolates have been obtained from sick goats and poultry, as well as from human patients with, for example, wound infections, endocarditis, and septic shock [28,29]. Less is known about the species *S. cohnii*. However, a few case reports confirm this CoNS as a possible source of infection in animals [26,30]. In a previous study from Switzerland, methicillin-resistant *S. cohnii* was identified as the primary pathogen in a dog with an urinary tract infection [26]. In another case series, *S. cohnii* was the causative agent of canine pyoderma in two out of 166 CoNS isolates (1.2%) [30]. To the best of the authors’ knowledge, *S. equorum* and *S. saprophyticus* have not been isolated from infectious diseases in dogs to date. However, methicillin-resistant strains have been identified on the mucous membranes of horses and humans, as well as in samples from stables [27]. In humans, *S. saprophyticus* is a common cause of urinary tract infections in young female patients and less commonly can be responsible for acute pyelonephritis, epididymitis, and prostatitis [31]. With respect to the current literature, at least three of the five CoNS, *S. haemolyticus*, *S. lentus*, and *S. cohnii,* isolated in this study have the potential to cause MDR infections in canine and human populations. The remaining two CoNS, *S. equorum* and *S. saprophyticus*, harbor the *mecA* gene, which may be relevant considering horizontal gene transfer. CoPS and CoNS carry conjugative plasmids, which can be transferred between different staphylococcal species. The previously mentioned genomic island *SCCmec* containing the *mecA* gene is frequently located on such plasmids, leading to a potential spread of methicillin-resistance between staphylococci species by plasmid transfer. CoNS like *S. epidermidis* are suspected to serve as a reservoir for *SCCmec* [32]. A possible horizontal transfer of *SCCmec* from *S. haemolyticus* to *S. aureus* has also been described [33]. Thus, although *S. equorum* and *S. saprophyticus* may not be relevant for infections in dogs, they could pose a potential health risk through *mecA* gene transfer to pathogenic staphylococcus species, although this rarely happens. In addition to assessing the infection risk of the various staphylococci isolates detected, the transmission between humans and animals must also be analyzed. Nocera et al. (2023) not only showed that owners maintain very close contact with their dogs, but also that the number of companion dogs has notably increased during the last years with more than 72 million households owning dogs in the European Union. In that study, it was possible to recover the same bacterial strain in two dogs and their owners. These included *S. epidermidis* and *S. aureus* showing overlapping phenotypes and similar antimicrobial resistance profiles. Although transmission was suspected, no genotypic testing was performed [20]. On the other hand, other studies observed that bacterial isolates taken from humans and their pets were genetically distinct in the majority of cases and that animal–human transmission may only play a minor role in the spread of MDR pathogens [18,34]. Nevertheless, the transmission of MRSA between humans and animals has been proven in the past and therefore, comprehensive microbiological surveillance and a multidisciplinary “One Health” approach to understand the route of infection is of great importance [18].

To date, only a few studies have focused on facilities such as DDFs or similar. Gould et al. (2020) [8] conducted a study in pet grooming salons in Washington (USA) and tested 12/19 (63.2%) salons positive for MRS (MRSP, MRSA, and MRSS). In our study, there was a comparatively lower number of MRS detected. However, these two studies did not measure the exact same criteria. [8] mainly investigated equipment that had direct contact with the animals’ skin such as grooming clippers, whereas our study focused on indirect contact with the interior of the facilities. In addition, the low number of participating facilities in both studies hinders exact comparisons. Two further studies investigated the prevalence of either *S. aureus* or multiple *Staphylococcus* species and *Enterobacteriaceae* in German animal shelters [35,36]. However, only [35] included environmental samples from door handles, the telephone receiver, seats, and other surfaces. Similarly to our study, MRS detection was poor. Out of 25 samples, only one MRSA isolate was detected from the floor of the quarantine box in which an MRSA-infected cat was previously housed. No further *S. aureus* strains were isolated [35]. There are some limitations regarding the comparability of these studies with our investigation. [36] sampled humans and animals, but collected no data about the bacterial load or presence of MDR bacteria in the environment. Additionally, the condition in animal shelters differs in some points from that of DDFs. One main aspect is that animal shelters often keep more than one animal species and that the animals often come from abroad, both of which potentially increase the abundance of pathogens. In terms of hygiene, however, there could be parallels to DDFs; this may still be more comparable to veterinary clinics, as also sick animals are kept in animal shelters and there is often a quarantine station. This enhances requirements on the cleaning and disinfecting of the environment and handling of the animals. To the authors’ knowledge, this is the first study investigating the presence of MDR bacteria in DDFs.

One major goal of the “Global Action Plan on Antimicrobial Resistance” of the WHO and the “One Health” approach is to foster an understanding of how AMR spreads in different settings [1]. In our study, the *mecA* gene was only detected in staphylococci species that are rarely associated with infections, mainly in immune-compromised patients. However, the possibility of transferring the *mecA* gene to pathogenic species and thus contributing to the spread of resistance cannot be excluded.

The second objective of this study was the investigation of DDFs concerning the presence of other opportunistic pathogens, which can cause serious infections in animals and humans. Members of the genus *Acinetobacter*, which are non-fermentative Gram-negative coccobacilli, ubiquitously present in the environment, are of emerging concern as they are known to cause severe infections and nosocomial outbreaks in veterinary and human clinics [13]. A total of 109 *Acinetobacter* isolates, belonging to 23 different *Acinetobacter* species, were detected in 93.8% of the DDFs (15/16 DDFs) in this study. These included 17 species of the *Acb* complex and eight *A. radioresistens* isolates harboring a carbapenemase gene. Members of the *Acb* complex are particularly associated with multidrug resistance due to a variety of mechanisms [11]. The main cause of carbapenem resistance in *A. baumannii* is the expression of acquired carbapenemases such as OXA-23. The encoding *bla*_OXA-23_ gene can either be located on the chromosome or on plasmids [10]. Especially, this *Acinetobacter* species is considered a high-risk pathogen in the WHO ranking of global priority pathogens associated with outbreaks involving high mortality rates [9,12,13]. A retrospective study described the death of 70% of the infected dogs and cats in a veterinary hospital, either due to infection or clinical deterioration, followed by euthanasia [12]. Our study revealed four isolates of *A. baumannii* on a dog bed, a bowl, leashes, and a door handle. The last two localizations in particular pose an increased risk of transmission to both dogs and humans. Thirteen more isolates belonging to the *Acb* complex were detected on multiple sample locations. *Acinetobacter calcoaceticus*, *A. pittii*, *A. nosocomialis*, and a few other newly added *Acinetobacter* species are phenotypically and genotypically closely related to *A. baumannii*. The definitive species identification of those without applying genomic sequencing approaches is challenging and therefore, these pathogens are grouped into the *Acb* complex [13]. In this study, they were isolated from seven DDFs (43.8%), with none of these isolates being positive for any of the investigated carbapenemase genes. However, as *Acinetobacter* spp. can develop AMR extremely rapid, their detection in facilities with a high animal population should raise concern about a possible route of infection [10].

By screening multiple *Acinetobacter* spp., [37] identified *A. radioresistens* as a natural source of the *bla*_OXA-23_ gene that has been mobilized from the chromosome of this species and can confer carbapenem resistance in *A. baumannii*. Here, carbapenemase genes located on plasmids enable easy transmission via conjugation [37]. In addition to sharing these genes, a small but increasing number of recent case reports have described infections with *A. radioresistens* in humans [38,39]. In veterinary medicine, the prevalence of *Acinetobacter* spp. has been investigated in canine and feline routine clinical samples, with only three *A. radioresistens* isolates identified in 125 *Acinetobacter* spp.-positive samples [11]. In our study, 8 of the 12 *A. radioresistens* isolates from three DDFs (18.8%) harbored the *bla*_OXA-23_ gene and therefore, may either pose a risk of infection or of transferring this gene to other *Acinetobacter* species.

No genotypic comparative investigations with nosocomial *Acinetobacter* strains have been performed in this study. However, *Acinetobacter* spp. isolates carrying the *bla*_OXA-23_ gene on the same plasmids as described for a human lineage have been identified in a veterinary hospital recently [21]. Clinical outbreaks of *A. radioresistens* or members of the *Acb* complex in DDFs have not been described so far. However, outbreaks of canine oral papillomavirus infection in DDFs and regional leptospirosis indicate that concentrated canine populations, the admission of animals from different backgrounds, and direct animal contact can create an ideal environment for pathogen transmission [40,41]. Therefore, the detection of carbapenemase-positive *Acinetobacter* spp. and *Acinetobacter* of the *Acb* complex in DDFs should be considered an animal health concern and also be addressed in terms of the “One Health” approach.

This study did not reveal any correlation between cleaning practices and the evidence of either MRS or carbapenemase-positive *Acinetobacter* spp. This is consistent with the findings of [8] for pet grooming salons. The questionnaire used in that study was completed by multiple people from each salon, and discrepancies in cleaning practices between operators and groomers were identified. In the DDFs, the questionnaire was completed exclusively by the owner of the facility, so no statements can be made about deviations in implementation by staff members. Currently, standardized cleaning protocols, as they exist for veterinary clinics, for example, are not available for DDFs or grooming salons [42]. This also applies to the standardization of cleaning products. However, one DDF reported that they had received precise clinic instructions from their regional veterinary office. Statistically, there were significantly more bacterial isolates detected on the dog bed and floor, which could be due to the increased contact of the dogs with these surfaces. Another explanation for the detection of pathogens could be an insufficient cleaning frequency or improper use of cleaning products. Furthermore, in some DDFs, cleaning was not always performed by the same person, which can lead to inconsistencies in cleaning regimes.

None of the examined pathogens with multidrug resistance genes could be isolated from DDFs where acetic acid, ADBAC, ethanol/ethyl-alcohol, or PermaZym^®^ technology was used. Various studies have shown an antimicrobial effect of all but one of these agents. Acetic acid, ethanol (or ethanol leaf extract), and ethyl-alcohol have even been shown to inhibit the growth of MRSA and *Acinetobacter baumannii* in vitro [43,44,45]. In contrast, it was shown that low ethanol concentrations significantly enhance the growth rate and cell density of *A. baumannii* and increase the expression of potential virulence functions [46]. Alkyl-dimethyl-benzyl-ammonium-chloride belongs to the earliest disinfectants within the family of quaternary ammonium compounds (quats). These have a wide spectrum of mechanisms of action against multiple microorganisms including Gram-positive and Gram-negative bacteria [47]. To the authors’ knowledge, there are no publications regarding the antibacterial efficacy of PermaZym^®^ technology used in Biodor (Biodor GmbH, Bocholt, Germany). The product contains microorganisms (class of risk 1), benzisothiazolinone, and bronopol. The latter two ingredients, however, are known for their antimicrobial properties [48,49,50]. In a recent study, the antimicrobial effect of bronopol was tested with multiple pathogens. The substance demonstrated low minimum inhibitory concentrations (MICs) against Gram-negative pathogens like *A. baumannii*, indicating a high antimicrobial efficacy against these species. A higher MIC was detected against Gram-positive bacteria like MRSA, suggesting lesser antimicrobial efficacy. However, other publications did not show a significant difference in efficacy between Gram-positive and Gram-negative species both for bronopol [50]. Dog daycare facilities in which MRS and carbapenemase-positive *Acinetobacter* spp. were detected used cleaning products containing lactic acid, surfactants, or phenoxyethanol/benzisothiazolinone. All of these agents have been tested for antimicrobial efficacy in various studies with a low MIC against Gram-negative and Gram-positive bacteria for lactic acid and phenoxyethanol and a biocidal effect on various bacteria and fungi for benzisothiazolinone [48,51,52]. Ref. [49] also achieved promising results with benzisothiazolinone-based inhibitors against *S. aureus* sortase A, an important cell wall enzyme. Surfactants are divided into four major classes (cationic, anionic, amphoteric, and nonionic). Studies confirm antimicrobial activity against *S. aureus* for anionics and amphoterics, and against *E. coli* for amphoterics and nonionics. Cationic surfactants, which include the previously described quats, show a high affinity for the interfaces of multiple bacteria (Gram-positive and Gram-negative) as well as viruses and fungi [47,53]. According to the author’s understanding, of these chemicals, only phenoxyethanol-based novel agents have been recently tested with a high inhibitory activity against MRSA [54].

In order to optimize the efficacy of cleaning regimes in DDFs and similar institutions with a high animal density and a close human–animal interaction environment, standardized cleaning protocols or guidelines would be of great benefit to DDF operators, owners, and their dogs.

A major limitation of this study is the small sample size. As only 16 DDFs could be included, and only a small number of *Staphylococcus* spp. isolates (n = 38) were detected, this influenced the statistical analysis. Although the included DDFs were all similar in terms of kennels and interior, it is possible that these facilities were not representative of the broader population of DDFs. There might also be a geographical variability, especially comparing urban and suburban regions. In order to obtain a broader overview, several regions in Germany were intentionally included in this study, as well as DDFs in the city and in the countryside. As a further limitation, the choice and availability of sample locations in the DDFs varied in setting and interior and a comprehensive comparison between the institutions could not be achieved. However, to improve data comparability, several sites were pooled, leading to five pooled and two non-pooled groups of sample locations for statistical analysis. This in turn could have diminished the role of individual sample sites but was considered negligible. Finally, locations such as the “floor” included samples from different textures like ceramic tiles, laminate, wooden floors, and carpets. The variety in surface materials of floors may have had an impact on results and evaluation. Another additional limitation of the study concerns the prediction of phenotypic AMR by PCR-based gene identification. A recent study showed a lack of accuracy for genotypic predictions as only a poor correlation between next-generation sequencing and culture-based antimicrobial susceptibility testing of *Staphylococci* spp. was observed [55]. However, other studies argue that the actual limitation of genotypic methods displays the incomplete knowledge of the genetic basis of resistance and consistent comparison to phenotypically investigated strain sets is necessary to maintain an accurate prediction of AMR [56,57,58]. It can, therefore, be assumed that the results of this study are phenotypically relevant.

## 5. Conclusions

In summary, methicillin-resistant staphylococci and carbapenemase-positive *Acinetobacter* (*A*.) *radioresistens* isolates were detected in dog daycare facilities. However, only a small number of DDFs were tested positive for these pathogens. In 25% (4/16) of the DDFs, 18 coagulase-negative, *mecA*-positive staphylococci isolates and in 18.8% (3/16) of the DDFs, eight *bla*_OXA-23_-positive *A. radioresistens* were detected, respectively. Though these isolates were not high-risk pathogens, they pose a risk of spreading AMR genes and a risk of transmission between humans, dogs, and the environment according to the “One Health” concept. *Acinetobacter calcoaceticus*, *A. baumannii*, and *A. pittii*, which have been described as opportunistic pathogens in human and veterinary medicine, were isolated in 43.8% (7/16) of the DDFs. These isolates, however, did not carry any of the AMR genes investigated. The dog daycare facilities also differed in their cleaning practices. Various cleaning products were used, often with presumed good antimicrobial effects as shown by previous experimental studies. This underlines the severity of the situation, as the previously mentioned bacteria were detected despite regular cleaning. Exceptions that showed a lack of MRS and MDR *Acinetobacter* detection and therefore a presumed good cleaning efficacy included the DDFs that used acetic acid, ADBAC, ethanol/ethyl-alcohol, or PermaZym^®^. A contaminated environment belongs to the key factors for pathogen transmission in veterinary clinics which have, like dog daycare facilities, a high animal population density. However, standardized cleaning protocols or guidelines, as they exist for clinics, are currently not available in DDFs and should be established to optimize the efficacy of cleaning procedures and prevent the spread of MDR pathogens.

## Figures and Tables

**Table 1:** Cleaning products used in dog daycare facilities (DDFs) in correlation with the frequency of isolated methicillin-resistant staphylococci (MRS) and carbapenemase-positive *Acinetobacter* species.

Product Name	Active Ingredient	Frequency of DDFs with Bacteria Isolated	
		MRS	Carbapenemase-Positive *Acinetobacter* spp.
Commercial universal cleaning agent	Surfactants	2	0
**Sagrotan (B Hygiene Home Deutschland GmbH, Heidelberg, Germany)** Prowin (proWIN Winter GmbH, Illingen, Germany)	Surfactants and Lactic acid	1	1
**Kärcher (Alfred Kärcher Vertriebs-GmbH, Winnenden, Germany)**	Phenoxyethanol and benzisothiazolinone	1	1
Vinegar cleaner	Acetic acid	0	0
Biguanid Surface N (Dr. Schumacher GmbH, Malsfeld, Germany)	Alkyl-dimethyl-benzyl-ammonium-chloride	0	0
Biodor (Biodor GmbH, Bocholt, Germany)	PermaZym^®^	0	0
Dr. Becher Rapid disinfection (Dr. Becher GmbH, Seelze, Germany)	Ethanol and Ethyl-alcohol	0	0

## Data Availability

The data presented in this study are contained within this manuscript and are available upon request from the corresponding author.

## Supplementary Materials

The following supporting information can be downloaded at: https://www.mdpi.com/article/10.3390/vetsci11110568/s1, Questionnaire about the details of the participating dog daycare facilities and their cleaning protocols.
