# Supplementary material for: Presence of Methicillin-Resistant Staphylococci and Carbapenemase-Positive Acinetobacter Isolates on Surfaces in German Dog Daycare Facilities and Correlation with Cleaning Practices
Source: Vet Sci. 2024 Nov 15;11(11):568. doi: 10.3390/vetsci11110568 (PMC11599097; doi:10.3390/vetsci11110568)
Supplement: Supplementary file 1 [file vetsci-11-00568-s001.zip › Supplementary Materials.pdf]

Questionnaire: Veterinary study in German dog daycare facilities

How many dogs can you accommodate? _______________

How many dogs are visiting your daycare every day on average? _______________

Most of the dogs are visiting the daycare…
☐ 5 days ☐ 4 days ☐ 3 days ☐ 2 days ☐ 1 day per week

Do the same dogs visit the daycare every day?
☐ Yes, there are always the same dogs depending on the day of the week
☐ No, there is a constant change

Are there constant groups of dogs? ☐ Yes ☐ No

What health data do you request before accepting a dog in your facility?
☐ Vaccination status
☐ Last application of flea and tick prevention
☐ Last application of deworming
☐ Pre-existing health conditions
☐ Country of import, if existing
Others: ____

What health requirements do you impose?
☐ Vaccination status up to date
☐ Regular flea and tick prevention - every 4-12 weeks depending on the agent
☐ Regular flea and tick prevention - no time specified
☐ Regular deworming - every 3-4 months
☐ Regular deworming - no time specified

When is a dog not permitted to visit?
☐ In case of vomiting and diarrhoea
☐ In case of acute skin issues
☐ After the stay in a veterinary clinic
☐ Others: ____

Do the owners bring their own food for the dogs?
☐ Yes
☐ Alternating
☐ No

Do the dogs get treats?
☐ Yes, we have treats in our facility
☐ Yes, but every dog brings its own treats
☐ No

Food and water bowls...
☐ Are shared by several dogs

☐ Each dog gets its own bowl

Dog beds…
☐ Are shared by several dogs
☐ Each dog gets its own dog bed

Cleaning practice:
Which product is used to clean food and water bowls? ________________________________

How often are food and water bowls cleaned? ____

Which product is used to clean surfaces and dog beds?

____

How often are surfaces and dog beds cleaned? ____

At what time is cleaning done?
☐ In the morning, before dogs arrive
☐ While dogs are at the facility
☐ In the evening, after all dogs leave the facility

Do you offer trips or pick-ups by car?
☐ Yes
☐ No

If yes, how often and with which product do you clean the transporting vehicle?

____

Thank you for your participation!

Stephanie Forbes
Veterinarian/Resident ECVD
AniCura Tierärztliche Spezialisten Hamburg
Stephanie.forbes@anicura.de

AniCura Tierärztliche Spezialisten Hamburg
Rodigallee 85
220 43 Hamburg | Germany
Telefon +49 40 88 88 854 0
Fax +49 40 88 88 854 20
www.anicura.de/tierarztliche-spezialisten-hamburg

In Cooperation with **Justus-Liebig-Universität Giessen**
